# Effects of dietary fiber on Chinese children with functional constipation and targeted modification of gut microbiota and related metabolites

**DOI:** 10.3389/fnut.2025.1579668

**Published:** 2025-07-14

**Authors:** Pei Xiao, Ting Song, Xiao Lu Li, Yong Mei Xiao, Rui Xue Wang, Fei Fei Song, Dan Li, Ai Hua Zhang, Ying Wang, Ting Zhang

**Affiliations:** ^1^Department of Gastroenterology, Hepatology and Nutrition, Shanghai Children’s Hospital, School of Medicine, Shanghai Jiao Tong University, Shanghai, China; ^2^Shanghai Jiao Tong University, Shanghai, China; ^3^Nanxiang Branch Ruijin Hospital, Shanghai, China; ^4^Division of Pediatric Gastroenterology and Nutrition, Xin Hua Hospital, School of Medicine, Shanghai Jiao Tong University, Shanghai, China; ^5^Institute of Pediatric Infection, Immunity and Critical Care Medicine, School of Medicine, Shanghai Jiao Tong University, Shanghai, China

**Keywords:** FC, dietary Fiber, gut microbiota, metabolites, children

## Abstract

**Background:**

Although dietary fiber is widely recommended for preventing and treating functional constipation (FC), clinical trial evidence remains limited and the efficacy has not been sufficiently tested in children.

**Purpose:**

This study aimed to evaluate the effects of dietary fiber on FC symptoms, while identifying modulations in gut microbiota and associated metabolic changes.

**Results:**

Between January 1, 2024, and June 1, 2024, a total of 60 patients diagnosed with FC were enrolled in the study across three centers; however, 54 children completed the study. The final cohort consisted of 28 boys and 26 girls, aged 6 to 12 years (mean age: 8.4 ± 1.8 years). Following the dietary fiber intervention, a significant increase in the frequency of complete spontaneous bowel movements (CSBMs) was observed, accompanied by improved stool consistency. Scores for abdominal pain, bloating, and straining showed significant reductions. After 4 weeks of dietary fiber treatment, both richness and diversity of gut microbiota were significantly enhanced. At the genus level, the relative abundances of *Lachnospiraceae_ND3007_group*, *Lactococcus*, *Prevotella*, and *Anaerofustis* significantly increased, whereas *Enterobacter*, *DTU089*, and *Sutterella* showed significant decreases. Metabolic analysis revealed significant profile alterations. Kyoto Encyclopedia of Genes and Genomes (KEGG) enrichment analysis identified metabolite-associated pathways, including steroid hormone biosynthesis, alpha-linolenic acid metabolism, and nucleotide metabolism. Pearson correlation analysis established correlations among dietary fiber, gut microbiota, metabolites, and constipation relief. No significant adverse effects were observed.

**Conclusion:**

In conclusion, our findings indicate that dietary fiber alleviates constipation and is accompanied by intervention-specific alterations in gut microbiota and metabolites. This research elucidates the interrelationships between constipation, gut microbiota, and metabolites. These insights may enhance our understanding of the pathogenic mechanisms of FC and provide novel therapeutic perspectives.

**Clinical trials registration:**

ChiCTR2400084125.

## Introduction

Functional constipation (FC) is defined by the infrequent elimination of hard stools, frequently accompanied by pain during bowel movements and potential fecal incontinence. The diagnosis of FC in children relies on the pediatric Rome IV diagnostic criteria (1, 2). This condition is among the most prevalent disorders related to gut-brain interaction in individuals aged 18 and younger (3), with a global prevalence ranging from 0.5 to 32.2% in pediatric populations (4). The symptoms associated with FC can significantly impact a child’s quality of life, potentially resulting in school absenteeism and considerable healthcare-related expenses. Effective intervention strategies are urgent to relieve the FC difficulties, particularly in child populations.

Non-pharmacological management, such as education, demystification, lifestyle adjustment, and toilet training, as the first step in the treatment of functional constipation (5). Pharmacological treatment for FC primarily involves the use of irritant laxatives, which are commonly employed to alleviate constipation symptoms. However, prolonged use of these medications can result in “laxative-dependent” constipation, which not only harms gastrointestinal tissue but also exhibits a high recurrence rate (6). Many recent research studies have focused on dietary fibers, which are non-digestible carbohydrates with a degree of polymerization of 3 or more monomeric units. Codex-defined fiber includes non-starch polysaccharides such as cellulose, hemicelluloses and pectins, resistant starch and non-digestible oligosaccharides such as inulin and oligofructose, as well as lignins (7). Dietary fibers have been demonstrated to have several important associations with the management of various diseases, as evidenced by both epidemiological and interventional studies (8–10).

This study aimed to evaluate the effectiveness of dietary fiber in alleviating constipation symptoms in Chinese pediatric patients diagnosed with functional constipation (FC). Additionally, we sought to identify alterations in gut microbiota and metabolites resulting from the dietary intervention, with the objective of identifying representative biomarkers of FC and examining the regulatory effects of dietary fiber on these biomarkers.

## Methods

### Study design and participants

This study was a self-controlled trial conducted from January 2024 to June 2024 across three centers, including Shanghai Children’s Hospital, Nanxiang Branch Ruijin Hospital, and Xin Hua Hospital. We recruited pediatric patients aged 6 to 12 years who met the Rome IV criteria for child and adolescent functional constipation (1), as determined by the investigator. The inclusion criteria were: meet two or more of the following conditions at least once a week for more than 1 month, with insufficient basis for a diagnosis of irritable bowel syndrome (IBS): (1) Defecation in the toilet ≤ 2 times per week; (2) fecal incontinence occurring at least once a week; (3) postures related to fecal retention or a history of significant fecal retention; (4) a history of painful or difficult defecation; (5) large fecal masses in the rectum; (6) thick fecal masses that have blocked the toilet bowl. Exclusion criteria included: (1) allergies or adverse reactions to Testa Triticum Tricum Purif or similar drugs; (2) intestinal obstruction; (3) irritable bowel syndrome; (4) use of prebiotics, probiotics, antibiotics, or laxatives within the past 4 weeks; (5) severe metabolic diseases or chronic conditions affecting the cardiovascular, cerebrovascular, pulmonary, hepatic, or renal systems; (6) mental and psychological disorders; (7) alcohol, toxin, and drug abuse; (8) participation in clinical trials of other investigational drugs within the past 12 weeks; (9) constipation resulting from other diseases, functional defecation disorders, or medications, such as rectocele, pelvic floor spasm, or megacolon; (10) long-term laxative abuse.

From January 2024 to June 2024, 120 individuals who completed the screening by the investigator through clinic or online, of which 30 were excluded for not meeting the inclusion criteria, too busy to participate, or other reasons. Finally, 60 participants were recruited. The study was approved by the Ethics Committee of Shanghai Children’ s Hospital, Shanghai Jiao Tong University (Approval number: 2023R049 - E03). Each participant or their parents provided written informed consent before the intervention. The present trial was registered at Chinese Clinical Trials Registry as ChiCTR2400084125.

### Intervention

All eligible subjects received 3.5 grams of Testa Triticum Tricum Purif twice daily for a duration of 4 weeks. The botanical source of Testa Triticum Tricum Purif is wheat bran. Through hydrolysis extraction, starch and phytic acid are removed while protein content is reduced, resulting in Testa Triticum Tricum Purif with total dietary fiber content 80%. The study comprised a total of five visits: a screening visit, a pre-intervention visit, visits at weeks 2 and 4 during the treatment period, and an end-of-study visit at week 6. Participants were required to visit the research centers at week 0 and 4 to collect intervention products and complete sample collection and case report forms. Upon collection, all fecal samples are immediately stored at −80°C. At weeks 2 and 6, the researcher conducted follow-up telephone calls. Participants were asked to maintain a daily stool diary throughout the intervention period, return all remaining intervention products at week 4, and report any adverse events.

### 16S rDNA sequencing

Bacterial DNA was extracted from fecal samples utilizing the QIAamp Fast DNA Stool Mini Kit (QIAGEN, Germany). To amplify the V3-V4 hypervariable regions of the bacterial 16S rRNA gene, primers 338F (5’-ACTCCTACGGGAGGCAGCAG-3′) and 806R (5’-GGACTAC HVGGGTWTCTAAT-3′) were employed in a thermocycler PCR system (GeneAmp 9,700, ABI, Foster, CA, United States). The resulting PCR products were extracted from a 2% agarose gel, purified using the AxyPrep DNA Gel Extraction Kit (Axygen Biosciences, Union City, CA, United States). Pair-end library was sequenced on an Illumina Noveseq 6,000 PE250 platform (Illumina Corporation, San Diego, CA, United States) by Shanghai Winnerbio Technology Co., Ltd. (Shanghai, China). The raw sequencing reads were demultiplexed, quality filtered by fastp (version 0.21.0), and merged by FLASH (version 1.2.7). The rarefaction analysis based on Mothur (version 1.30.1) was conducted to reveal the *α*-diversity indices, including the coverage, richness (Chao1), and diversity (Shannon) indices. UniFrac was used for *β*-diversity analysis. The characterization of microorganismal features differentiating the microbiota was performed using the linear discriminant analysis (LDA) effect size (LEfSe) method, which emphasizes both statistical significance and biological relevance. Redundancy analysis (RDA) was used to evaluate the influence of confounding factors on the composition of the microbiome using vegan and ggplot2 packages in R software (version 3.6.3).

### Metabolic profiling by UHPLC-Q-Exactive Orbitrap MS

Following slow thawing of samples at 4°C, 25 mg aliquots were weighed and transferred to 1.5 mL Eppendorf tubes. To each tube, 800 μL of pre-cooled (−20°C) extraction solvent (methanol:acetonitrile:water = 2:2:1, v/v/v) and 10 μL of internal standard were added. Two steel beads were introduced, and samples were homogenized using a tissue homogenizer (50 Hz, 5 min), followed by 10 min of ultrasonic bath treatment at 4°C. After 1 h of incubation at −20°C, centrifugation was performed at 25,000 rpm (4°C, 15 min). A 600 μL aliquot of the supernatant was collected and dried using a freeze vacuum concentrator. The residue was reconstituted with 600 μL of reconstitution solution (methanol: H₂O = 1:9, v/v), vortex-mixed for 1 min, and subjected to another 10 min ultrasonic bath treatment at 4°C. A final centrifugation step (25,000 rpm, 4°C, 15 min) was conducted, and the resulting supernatant was transferred to injection vials.

Metabolites in pretreated fecal samples were separated and detected by ultra-high performance liquid chromatography (UHPLC) (Vanquish UHPLC, Thermo) coupled to a Orbitrap (Q Exactive HF-X/ Q Exactive HF) in Shanghai Winnerbio Technology Co., Ltd. (Shanghai, China). In both ESI positive and negative modes, the mobile phase contained A = 25 mM ammonium acetate and 25 mM ammonium hydroxide in water and B = acetonitrile. The gradient was 98% B for 1.5 min and was linearly reduced to 2% in 10.5 min, and then kept for 2 min, and then increased to 98% in 0.1 min, with a 3 min re-equilibration period employed.

### Statistical methods

In this study, statistical analyses were performed using GraphPad Prism 9.0 (GraphPad Software, USA). Descriptive data are presented as mean ± SD for continuous variables. The Wilcoxon rank sum test was employed to compare changes in constipation symptoms before and after the intervention. Additionally, Pearson correlation analysis was utilized to assess the relationships between changes in constipation symptoms and alterations in other variables throughout the intervention. *p*-value of less than 0.05 was considered statistically significant.

## Results

### Participants and characteristics

A total of 60 patients were enrolled in this study; however, only 54 children completed it, and 51 provided biological samples. Six children withdrew from the study prior to its completion due to difficulties with the protocol, while three children did not provide biological samples. Demographic and baseline characteristic data are presented in Table 1. The participants, aged between 6 and 12 years, included 26 females (48.1%) and 28 males (51.9%). The mean age of the participants was 8.4 ± 1.8 years, and the mean duration of chronic constipation was 3.9 ± 2.8 years. At baseline, the mean frequency of complete spontaneous bowel movements (CSBMs) per week was 2.2 ± 1.1. Stool consistency was assessed using the 7-point ordinal pediatric Bristol Stool Form Scale (BSFS), with a mean score of 1.4 ± 0.6. The mean scores for abdominal pain, abdominal bloating, and straining were 0.9 ± 0.8, 0.7 ± 0.8, and 0.7 ± 0.9, respectively. Notably, 63.0% of the participants experienced fewer than three defecations per week.

**Table 1:** Baseline characteristics of the participants.

Category	Results (N = 54)
Characteristic	
Gender, male, n (%)	28 (51.9)
Age, years	8.4 ± 1.8
BMI, kg/m^2^	16.7 ± 2.7
Constipation evolution time, years	3.9 ± 2.8
Efficacy variables	
Frequency of CSBMs per week	2.2 ± 1.1
Stool consistency^⁎^	1.4 ± 0.6
Abdominal pain, score^⁑^	0.9 ± 0.8
Abdominal bloating, score^⁕^	0.7 ± 0.8
Straining, score^⁖^	0.7 ± 0.9
Less than 3 defecations per week, %	63.0

CSBMs: complete spontaneous bowel movements. Data are mean ± SD or n (%). * Based on the 7-point ordinal paediatric Bristol Stool Form Scale (1 = small hard lumps or balls, like pebbles; 2 = fat, sausage-shaped but lumpy and hard; 3 = like a sausage but with cracks on its surface; 4 = like a sausage or snake, smooth, and soft; 5 = like chicken nuggets, in soft smooth blobs; 6 = like oatmeal, in fluffy, mushy pieces; and 7 = like a milkshake, watery); ^⁑^ Based on responses to the following questions: Did your tummy hurt at all? (Yes or no.) If yes, how much did your tummy hurt? (1 = a tiny bit; 2 = a little; 3 = some; 4 = a lot); ^**⁕**^ Based on responses to the following questions: Did your tummy feel big and full? (Yes or no.) If yes, how big and full did your tummy feel? (1 = a tiny bit; 2 = a little; 3 = medium; 4 = very); ^⁖^ Based on responses to the following question: When you pooped, how hard did you push? (0 = not hard at all; 1 = I pushed a tiny bit hard; 2 = I pushed a little hard; 3 = I pushed hard; 4 = I pushed very hard).

### Evaluation of dietary fiber on constipation symptoms

**Figure 1:**
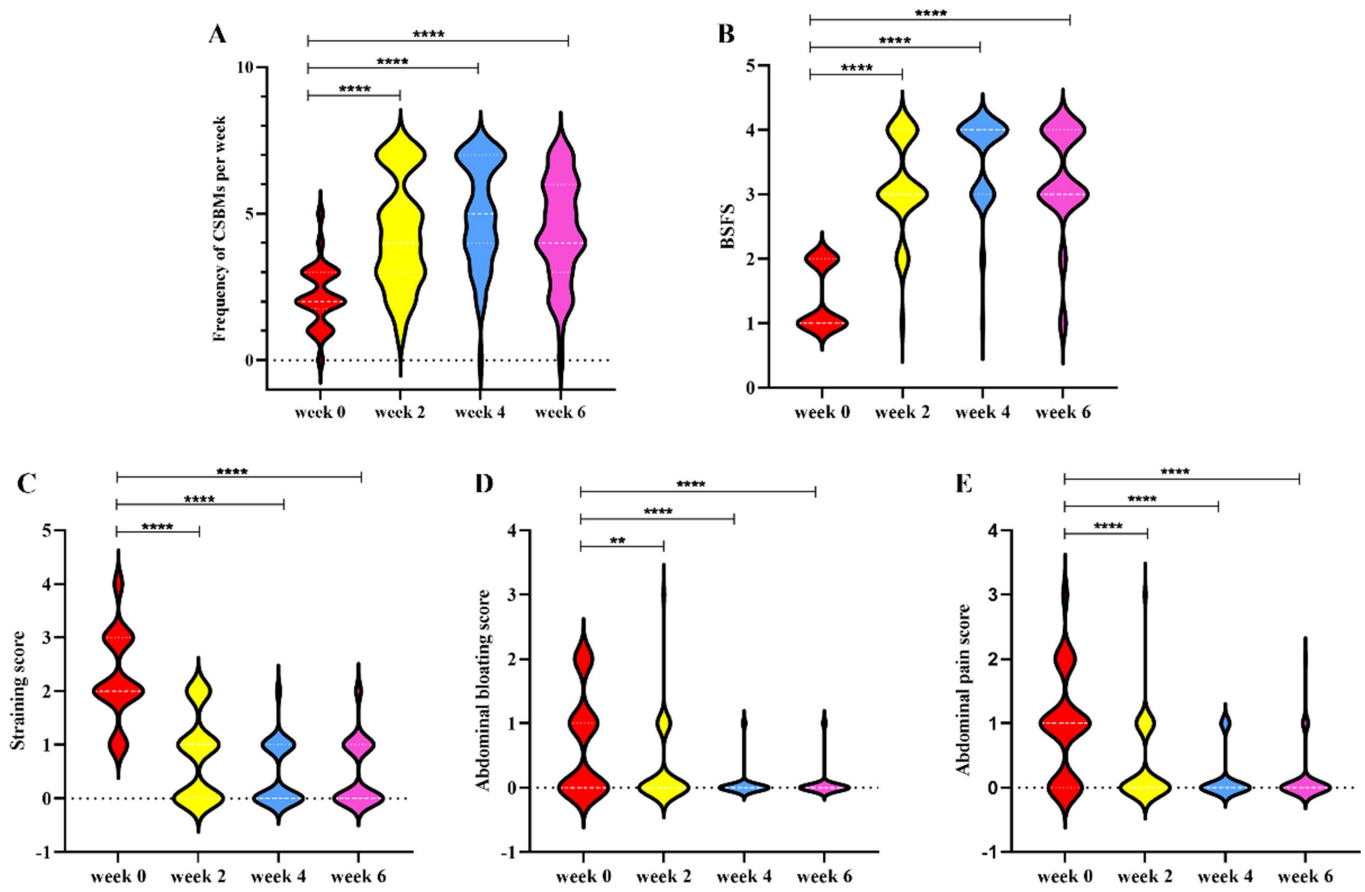
The changes of constipation-related indexes from baseline to week 2, 4, and 6. **(A)** Frequency of CSBMs per week; **(B)** BSFS; **(C)** Straining score; **(D)** Abdominal bloating score; **(E)** Abdominal pain score. Data are means with standard error. All detailed statistics can be found in Table 2. Significance was determined by using Wilcoxon rank-sum test. *p < 0.05, **p < 0.01, and ***p < 0.001. CSBMs: complete spontaneous bowel movements; BSFS: Bristol Stool Form Scale.

Following the intervention of dietary fiber, the frequency of CSBMs per week significantly increased at weeks 2, 4, and 6 (2 weeks post-treatment) compared to baseline levels, with statistically significant differences noted (Figure 1A). Additionally, stool consistency exhibited significant improvement at weeks 2, 4, and 6, also accompanied by statistically significant differences (Figure 1B). Furthermore, the scores for abdominal pain, abdominal bloating, and straining showed significant reductions at weeks 2, 4, and 6. These findings suggest that dietary fiber exerts a considerable positive impact on the symptoms of FC (Figures 1C–E). Comprehensive statistical details are provided in Table 2.

**Table 2:** Constipation symptoms over the intervention by week 2, 4 and 6.

Efficacy variables	Week 0	Week 2	Week 4	Week 6
Frequency of complete spontaneous bowel movements per week	2.2 ± 1.1	4.3 ± 1.8	5.0 ± 1.8	4.4 ± 1.7
Stool consistency^⁎^	1.4 ± 0.6	3.2 ± 0.7	3.6 ± 0.7	3.3 ± 0.8
Abdominal pain, score^⁑^	0.9 ± 0.8	0.3 ± 0.6	0.2 ± 0.4	0.1 ± 0.4
Abdominal bloating, score^**⁕**^	0.7 ± 0.8	0.3 ± 0.6	0.06 ± 0.2	0.06 ± 0.2
Straining, score^⁖^	0.7 ± 0.9	0.3 ± 0.5	0.07 ± 0.3	0.06 ± 0.2
Less than 3 defecations per week, %	63.0	16.7	5.6	3.7

Week 0 (baseline, *n* = 60), week 2 (Treatment for 2 weeks, *n* = 54), week 4 (Treatment for 4 weeks, *n* = 54), week 6 (2 weeks post-treatment, *n* = 54). Data are mean ± SD or n (%). * Based on the 7-point ordinal pediatric Bristol Stool Form Scale (1 = small hard lumps or balls, like pebbles; 2 = fat, sausage-shaped but lumpy and hard; 3 = like a sausage but with cracks on its surface; 4 = like a sausage or snake, smooth, and soft; 5 = like chicken nuggets, in soft smooth blobs; 6 = like oatmeal, in fluffy, mushy pieces; and 7 = like a milkshake, watery); ^⁑^ Based on responses to the following questions: Did your tummy hurt at all? (Yes or no.) If yes, how much did your tummy hurt? (1 = a tiny bit; 2 = a little; 3 = some; 4 = a lot); ^**⁕**^ Based on responses to the following questions: Did your tummy feel big and full? (Yes or no.) If yes, how big and full did your tummy feel? (1 = a tiny bit; 2 = a little; 3 = medium; 4 = very); ^⁖^ Based on responses to the following question: When you pooped, how hard did you push? (0 = not hard at all; 1 = I pushed a tiny bit hard; 2 = I pushed a little hard; 3 = I pushed hard; 4 = I pushed very hard).

### Effects of dietary fiber on gut microbiota diversity and composition

**Figure 2:**
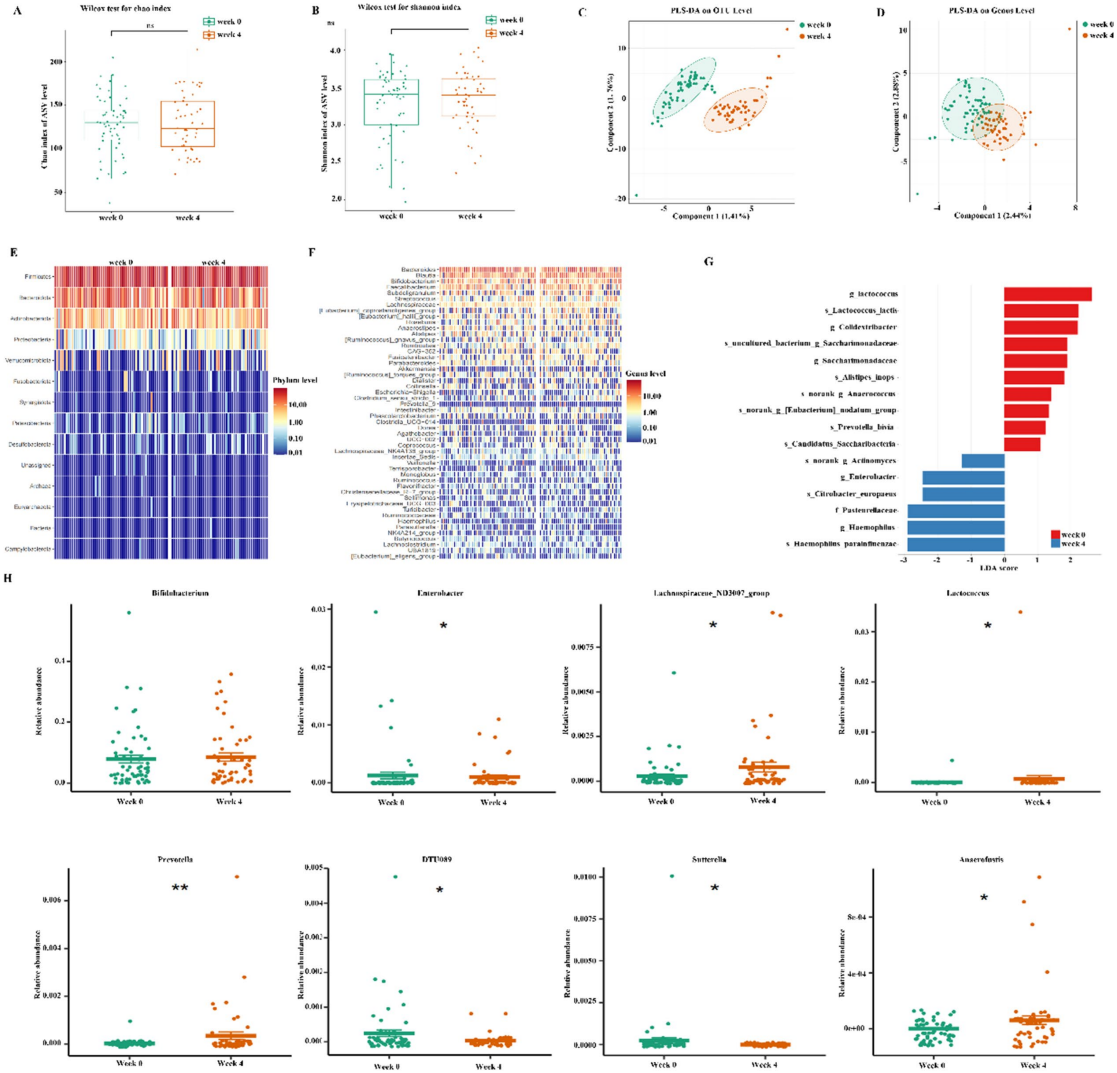
Effects of dietary fiber on gut microbiota. **(A, B)** Microbial richness indicated by chao1 index and Shonnon index; **(C,D)** Beta-diversity assay by Partial Least Squares Discriminant Analysis (PLS-DA); **(E, F)** Heatmap showed Microbiota composition at phylum and genus level; **(G)** LEfSe detected the taxa with the most significant differences in abundance of bacteria between the two groups. Bacteria with an LDA score greater than 3.5 were graphed. **(H)** The significant genus-level in abundance of bacteria between the two groups. Significance was determined by using Wilcoxon rank sum test. *p < 0.05, **p < 0.01, and ***p < 0.001.

The fecal microorganisms were assessed using 16S rDNA amplicon sequencing. A total of 2,095 operational taxonomic units (OTUs) were identified. The alpha diversity indices, specifically the Chao index and Shannon index, exhibited no significant differences between week 0 and 4 (Figures 2A,B). In the beta diversity analysis, the Partial Least Squares Discriminant Analysis (PLS-DA) plot indicated a clear separation between week 0 and week 4 at OUT and genus level, demonstrating significant differences in the gut microbiota following treatment with dietary fiber (Figures 2C,D). Additionally, we evaluated the differences in the abundance of bacterial communities at both the phylum and genus levels between week 0 and 4 (Figures 2E,F). LEfSe analysis revealed dominant bacterial communities, as illustrated by the histogram of LDA value distribution (LDA > 3.5, Figure 2G).

Subsequently, we examined the variations in gut microbiota between weeks 0 and 4 at both the phylum and genus levels. At the phylum level, we observed an increase in the relative abundances of *Firmicutes*, *Actinobacteriota*, and *Verrucomicrobiota* by week 4, whereas *Bacteroidota*, *Proteobacteria*, and *Synergistota* exhibited a decrease (Supplementary Figure S1). At the genus level, following the dietary fiber intervention, the relative abundances of *Lachnospiraceae_ND3007_group*, *Lactococcus*, *Prevotella*, and *Anaerofustis* significantly increased by week 4. In contrast, the abundances of *Enterobacte*r, *DTU089*, and *Sutterella* exhibited a significant reduction. Although the abundance of *Bifidobacterium* increased by week 4, this change was not statistically significant (Figure 2H).

### Altered gut metabolites associated with dietary fiber

**Figure 3:**
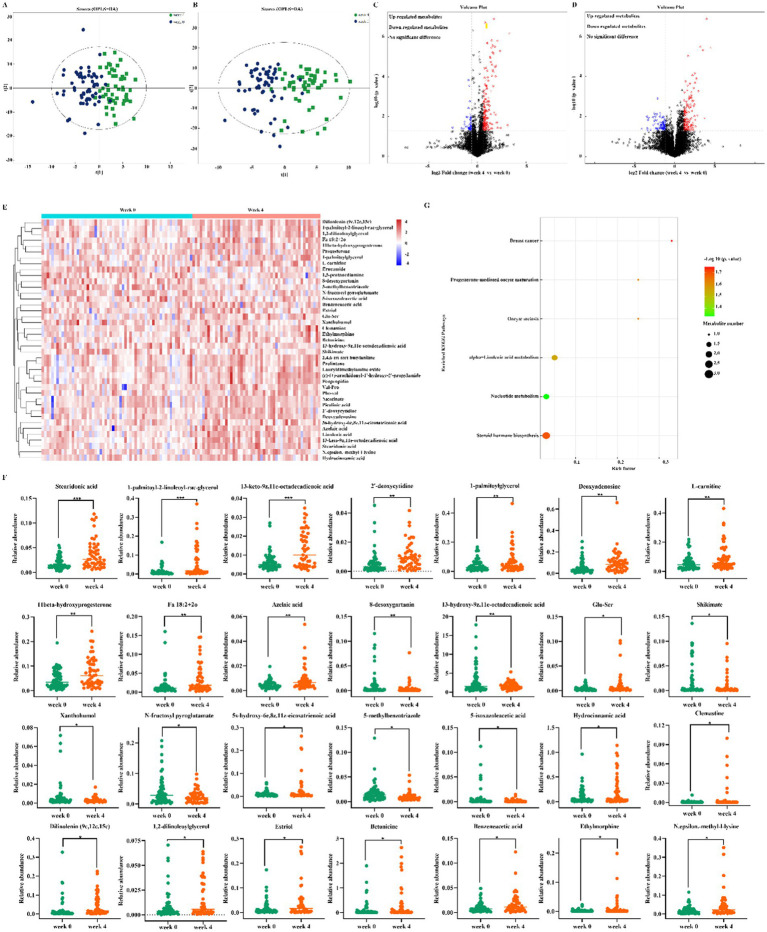
Altered gut metabolites. **(A,B)** OPLS-DA score plot of week 0 and 4 in the positive ion **(A)** and negative ion **(B)**. The differential metabolites were screened out according to the variable importance in projection (VIP) ≥ 1 and *p*-value <0.05; **(C,D)** Volcano plot of the significantly differential gut metabolites in the positive ion **(C)** and negative ion **(D)**. The bule color of the dot represents down, the red color of the dot represents down and black color of the dot represents no significant difference. **(E)** Heatmap analysis of the differential metabolites. **(F)** The significantly altered metabolites. **(G)** Bubble diagram of KEGG pathway enrichment analysis.

Gut microbiota actions are closely linked to the host microbial metabolic axis, for which metabolomics is a useful tool to reveal the interactions between the host and the gut microbiota. To identify characteristic metabolites between the two groups, untargeted metabolomics was performed. A total of 2,177 metabolites were identified, including 1,119 in positive ion mode and 1,058 in negative ion mode. The significant difference of metabolites between week 0 and 4 was screened by combining the variable importance in projection (VIP) value and the *p* value. VIP ≥ 1 and *p* < 0.05 are the common screening standards for differential metabolites among different comparison groups. Orthogonal partial least squares discriminant analysis (OPLS-DA) score plots displayed that there was clear separation between the two groups in both positive and negative ion modes (Figures 3A,B), which supports the possibility that dietary fiber regulates the metabolism of the intestinal microbiota. The 200 permutation test results verified that the OPLS-DA models were not overfitting. Volcano plots summarized the results of significantly differential up-regulated and down-regulated metabolites [Fold change (FC) > 1.5 or FC < 0.67 and *p*-value < 0.05] (Figures 3C,D). Heat maps were drawn, which could directly show the differences between the two differential group metabolites (Figure 3E). A total of 28 differential metabolites, which consisted mainly of organic acids, lipids, amino acids, fatty acids and nucleotides, were finally identified (Figure 3F). Kyoto Encyclopedia of Genes and Genomes (KEGG) enrichment analysis was performed to identify the pathways related to the different metabolites. Based on 28 differential metabolites, 5 metabolic pathways were obtained including steroid hormone biosynthesis, alpha-linolenic acid metabolism, nucleotide metabolism, breast cancer, progesterone-mediated oocyte maturation and oocyte meiosis, in which 3 metabolic pathways were selected as the most important metabolic pathways that were related to metabolic disturbances (Figure 3G). These metabolic pathways included steroid hormone biosynthesis, alpha-linolenic acid metabolism, nucleotide metabolism included. These findings indicated that these pathways might be involved in the anti-constipation effect of dietary fiber.

### Correlations among specific gut microbes, gut metabolites, and constipation-related indexes

**Figure 4:**
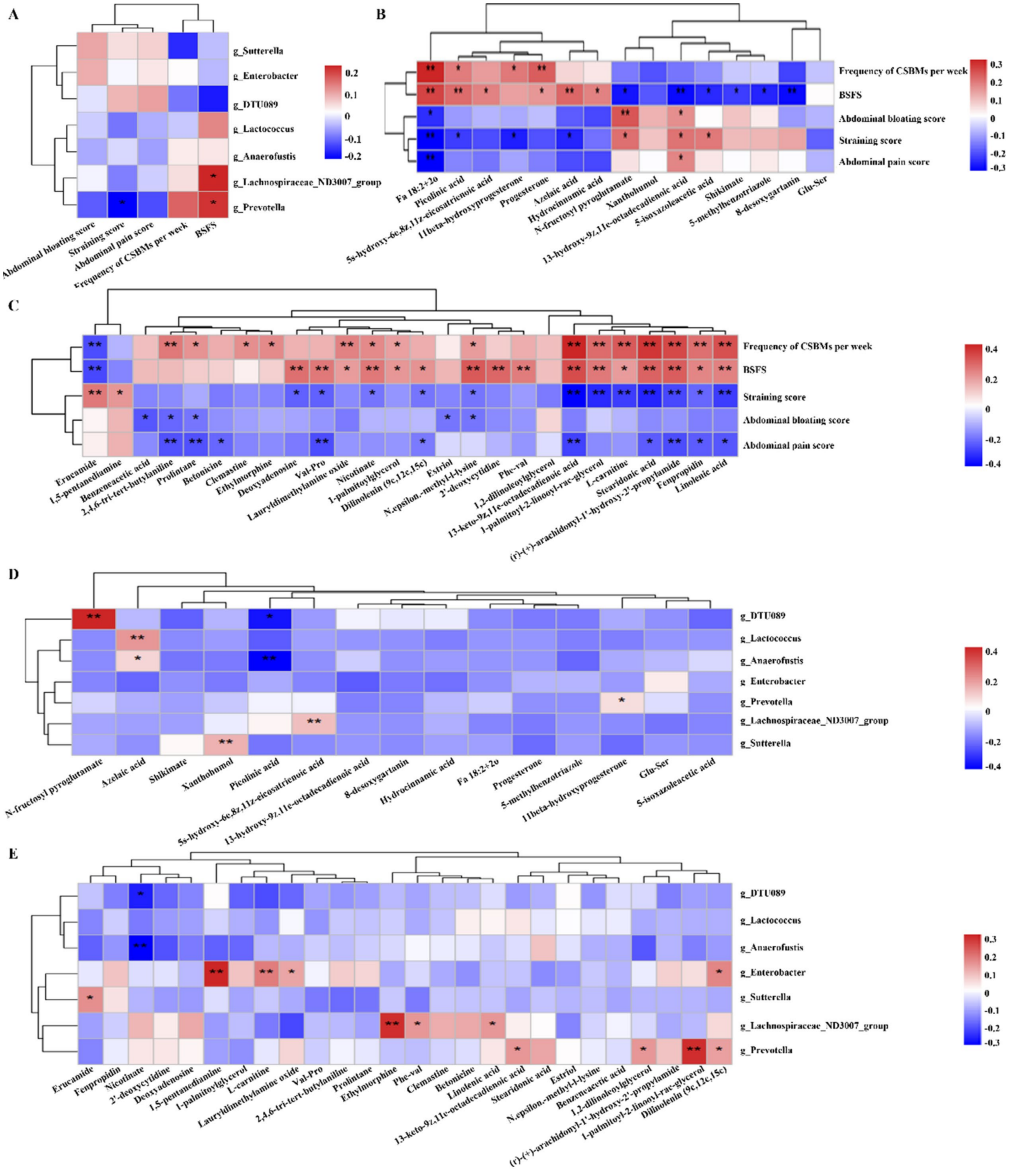
Heatmaps showing correlations between gut microbes, gut metabolites, and constipation-related indexes. **(A)** Correlations between the differential microbe taxa and constipation-related indexes. **(B,C)** Correlations between differential gut metabolites and constipation-related indexes in the positive ion **(B)** and negative ion **(C)**. **(D,E)** Correlations between the differential microbe taxa and metabolites in the positive ion **(D)** and negative ion **(E)**. The color at each intersection indicates the value of the r coefficient; *p < 0.05, **p < 0.01.

To elucidate the relationships among the factors contributing to the improvement of constipation by dietary fiber, Pearson correlation analysis (11) was carried out and the correlation coefficient heatmap was obtained. The correlations between differential gut microbiota and constipation-related indexes are illustrated in Figure 4A. *Prevotella* displayed a negative correlation with the straining score, whereas both Prevotella and *Lachnospiraceae_ND3007_group* demonstrated a positive correlation with BSFS.

Correlations between differential gut metabolites and differential constipation-related indexes are shown in Figures 4B,C. In the steroid hormone metabolism pathway of the negative ion mode, our findings indicate that 11beta-hydroxyprogesterone is positively correlated with frequency of CSBMs per week and negatively correlated with straining score; Progesterone is positively correlated with frequency of CSBMs per week and BSFS (Figure 4B). In the positive ion mode (Figure 4C), the metabolic pathways of alpha-linolenic acid, specifically stearidonic acid and linolenic acid, demonstrate a positive correlation with the frequency of CSBMs per week as well as with the BSFS. In contrast, these pathways exhibit a negative correlation with the straining score. Furthermore, nucleotide metabolism pathway, including deoxyadenosine and 2′-deoxycytidine, showed positively correlation with the BSFS. However, deoxyadenosine showed negatively correlated with straining score.

Correlations between the gut microbiota and gut metabolites are showed in Figures 4D,E. *Prevotella* exhibits a positive correlation with 11beta-hydroxyprogesterone within the steroid hormone metabolism pathway, while the *Lachnospiraceae_ND3007_group* shows a positive correlation with linolenic acid in the *α*-linolenic acid metabolism pathway. The close relationship between these differential metabolites and microbes implies that they may play an important synergistic role in the dietary fiber laxative process.

### Safety

No adverse effects (AEs), such as a new onset of abdominal pain, bloating, abdominal distension, excessive gas, diarrhea, or anaphylactic symptoms, were reported during the 4-week treatment with dietary fiber.

## Discussion

FC are burdensome, affecting quality of life and leading to absenteeism from school, and can also affect families (12). The existing understanding of the causes of FC encompasses issues related to intestinal motility, problems with intestinal secretion, alterations in visceral sensitivity, dysfunction of the pelvic floor muscles, and abnormalities in the enteric nervous system, among others (2). From the viewpoint of primary care practice, insufficient physical activity and inadequate consumption of water and dietary fiber are widely acknowledged risk factors for constipation (7). In children without underlying health issues who experience constipation, individual fiber supplements such as glucomannan, partially hydrolyzed guar gum, bran, and cocoa husk have been shown to increase stool frequency compared to a placebo group. Furthermore, glucomannan was associated with improved stool consistency, reduced abdominal discomfort, and a decrease in the frequency of painful defecation episodes (13–15). Research examining combinations of dietary fibers also demonstrated enhancements in constipation, an increase in daily bowel movements, and improved stool consistency (16, 17). Additionally, the impact of supplementing with extracted fibers on constipation-related issues differed, which can be partially attributed to variations in fiber type, amount, and the length of the intervention (7). Increasing evidence suggests that disturbances in gut microbiota play a pathological role in functional constipation. The main characteristics of gut microbiota in FC patients are the relative decrease of beneficial bacteria such as Lactobacillus and Bifidobacterium, the relative increase of potential pathogens, and the reduced species richness. Furthermore, gut microbiota can influence gut functions through the production of metabolites, including short-chain fatty acids (SCFAs) and bile acids (18). The Testa Triticum Tricum Purif employed in this study represent a commercially available dietary fiber supplement with distinct physicochemical properties compared to commonly investigated fibers (e.g., inulin, pectin, *β*-glucans). As a predominantly insoluble fiber, it exhibits superior water-holding capacity and slower fermentation kinetics compared to soluble counterparts. Unlike highly processed synthetic fibers, its natural lignocellulosic matrix preserves botanical microstructure, potentially modulating gut microbiota through both bulk-forming effects and gradual release of fermentable substrates. These unique properties position Testa Triticum Tricum Purif as a structurally intact, dose-titratable fiber source particularly suitable for chronic management of functional constipation. In this self-controlled trial involving children with FC, we observed a reduction in hard stools, abdominal pain, and abdominal bloating, as well as a decrease in straining. Additionally, the frequency of CSBMs per week increased. These results indicate that dietary fiber had a substantial positive effect on the symptoms of FC. To our knowledge, this is the first human trial investigating Testa Triticum Tricum Purif that has identified specific intervention-related changes with the positive effect of constipation.

The gut microbiome plays a critical role in maintaining health and contributes to disease progression. Although studies have demonstrated that the composition of the gastrointestinal microbiome significantly differs between individuals with constipation and those without (18, 19), the evidence regarding the influence of specific strains on the occurrence of constipation is often contradictory. An animal study (20) demonstrated that the abundance of *Phascolarctobacterium*, *Prevotella*, *Treponema*, *Butyricimonas*, *Bacteroides*, and *Lactobacillus* was selectively increased by different types of dietary fibers. Conversely, the abundance of *Clostridium perfringens* and *Bacteroides fragilis* was decreased by fibers. Khalif et al. (21) demonstrated that the levels of *Bifidobacteria*, *Lactobacilli*, *Bacteroides* and *Clostridium* species were decreased in FC patients, while the levels of *Enterobacteriaceae*, *Staphylococcus aureus* and fungi were increased. In an adult study (22), *Anaerostipes* showed increasing trends after dietary fiber supplementation while this increasing trend tended to correlate with the increase in bowel movement frequency. In a bacterial experiment, the commensal *Anaerostipes* demonstrated its ability to transform dietary inositol into propionate and acetate (23), which are SCFAs that could lower intestinal pH, enhance colonic motility, inhibit pathogens (7). A recent study (24) found gut microbiota can influence the onset of constipation, while constipation can alter the gut microbiota. Notably, *Coprococcus1*, *Coprococcus3*, *Desulfovibrio*, *Flavonifractor*, and *Lachnospiraceae UCG004* play a protective role against constipation, whereas *Ruminococcaceae UCG005*, *the Eubacterium nodatum group*, *Butyricimonas*, and *Bacteroidetes* are associated with an increased risk. The conflicting data on the microbial alterations of patients with constipation may not only be attributable to age-related differences and certain individual differences but also to differences in DNA extraction methods (25). In our study, the relative abundances of *Lachnospiraceae_ND3007_group*, *Lactococcus*, and *Anaerofustis* were significant increased at week 4, while *Enterobacter* and *Sutterella* were significant reduced after dietary fiber intervention.

Normal defecation depends not only on adequate gut motility but also on the proper functioning of intestinal secretion (26). Alterations in the transport of fluids and electrolytes within the intestine represent a significant pathophysiological disturbance associated with constipation, which is further influenced by gut microbiota (27). Research has demonstrated that constipation-induced dysbiosis results in an increased water-retaining capacity of the colon, accompanied by a decrease in fecal water content (28). The gut microbiota can regulate the expression of aquaporins. Vandeputte et al. (29) demonstrated that the *Prevotella* (P) enterotype is more abundant in individuals with loose stools, whereas the *Ruminococcaceae-Bacteroides* (RB) enterotype predominates in samples from individuals with harder stools. The *Prevotella* enterotype is believed to enhance fecal water content and accelerate gut transit. In our study, the relative abundances of *Prevotella* was significant increased at week 4, while *DTU089* was significant reduced after dietary fiber intervention. These findings may illuminate the possible mechanisms by which dietary fiber alleviates constipation, specifically through optimizing gut microbes. Correlation analysis revealed that *Prevotella* exhibited a negative correlation with the Straining score, while both *Prevotella* and the *Lachnospiraceae_ND3007_group* showed a positive correlation with the Bristol stool form scale. Collectively, dietary fiber may significantly alleviate constipation through its interactions with gut microbes in patients with FC. Additionally, host gut microbiota may interact with dietary fibers, thus affecting microbiome metabolism and inhibiting the growth of pathogens (7, 30).

Dysregulation of the gut microbiota and its associated metabolites plays a crucial role in the progression of FC. However, the available literature has mainly focused on the pathogenesis of FC concerning gut microbiota, while less attention has been given to how the microbiome and metabolites interact in the context of FC development. The pathway changes were directly related to the ameliorative effect of dietary fiber on constipation. This study screened differential metabolites using OPLS-DA and volcano map, and KEGG enrichment analysis was performed to identify the pathways related to the different metabolites, revealing steroid hormone biosynthesis and alpha-linolenic acid metabolism as novel biomarkers for pediatric functional constipation. Studies previously demonstrated that the steroid hormone biosynthesis pathway could affect the internal intestinal environment, intestinal homeostasis, and metabolism (31). Steroid hormone signaling may be essential in maintaining colonic homeostasis, but additional research is necessary to comprehend its function. Increased levels of progesterone cause alterations in community structure, Nuriel-Ohayon et al. (32) demonstrate an increase in the relative abundance of Bifidobacterium in the 3rd trimester of pregnancy in both humans and mice, as well as in models of progesterone supplementation, indicating that progesterone contributes to the changes in the gut microbial community. Various studies have shown the significance of sex steroid hormones, such as testosterone and dehydroepiandrosterone, in regulating processes and signaling interactions along the brain-gut axis. This axis plays a crucial role in gut function and motility, further supporting gut homeostasis (33, 34). A recent study (35) found that supplementation with Xylooligosaccharides in the diet improved bowel function, gastrointestinal motility, serum regulatory peptides, and neurotransmitter levels in constipated mice. Xylooligosaccharides may exert anti-constipation effects through pathways associated with steroid hormone biosynthesis and alpha-linolenic acid metabolism. Our metabolomics findings indicate that the treatment of constipation with dietary fiber is similarly linked to steroid hormone biosynthesis and alpha-linolenic acid metabolism, which is consistent with existing literature.

*α*-Linolenic acid, an *ω*-3 polyunsaturated fatty acid, was an indispensable fatty acid that could only be obtained via diet rather than being manufactured by the human body. α-Linolenic acid could promote gastrointestinal motility in cecum resected rats (36), reduce inflammation of the colonic mucosa, and increase the abundance of bacteria associated with the intestinal mucosa (37, 38). Moreover, alpha-linolenic acid metabolism has been shown to improve constipation relief significantly (39). Rhubarb, a traditional herb, has been utilized in clinical practice for centuries to treat constipation. Yang et al. (11) found that rhubarb could improve the composition of intestinal microbiota in constipated rats. Specifically, beneficial bacteria such as Ligilactobacillus, Limosilactobacillus, and Prevotellaceae UCG-001 were significantly increased, while pathogens such as Escherichia-Shigella were notably decreased. Additionally, metabolites such as α-linolenic acid was markedly elevated in constipated rats. Consequently, the variations in the levels of these metabolites could be attributed to dietary fiber regulation of the steroid hormone biosynthesis pathway and alpha-linolenic acid metabolism, which may have a role in alleviating constipation.

Despite yielding meaningful findings, this study has several limitations that warrant consideration. Firstly, the single-arm design without a control group and the relatively small sample size may limit the generalizability of our conclusions, necessitating larger randomized controlled trials for validation. Secondly, the short follow-up duration precludes assessment of long-term intervention effects. Thirdly, the lack of sub-categorization based on constipation subtypes hindered differential effect analysis across pathophysiological classifications. Furthermore, while we identified global changes in gut microbiota composition and metabolomic profiles, the study did not perform in-depth characterization of specific microbial taxa or conduct targeted metabolic pathway analyses. Future investigations incorporating multi-omics approaches and mechanistic studies would help elucidate the precise interactions between dietary fibers and host-microbiota metabolism.

## Conclusion

In conclusion, our findings suggest that dietary fiber may alleviate constipation, accompanied by intervention-specific alterations in gut microbiota. Our research elucidates the correlation among constipation, gut microbiota, and metabolites. This study indicates that Testa Triticum Tricum Purif presents a novel potential for use as a functional diet or as a new therapeutic approach for patients with FC.

## Data Availability

The raw data supporting the conclusions of this article will be made available by the authors, without undue reservation.
