# Supplementary figures and images for: Effects of dietary fiber on Chinese children with functional constipation and targeted modification of gut microbiota and related metabolites
Source: Front Nutr. 2025 Jul 14;12:1579668. doi: 10.3389/fnut.2025.1579668 (PMC12301205; doi:10.3389/fnut.2025.1579668)

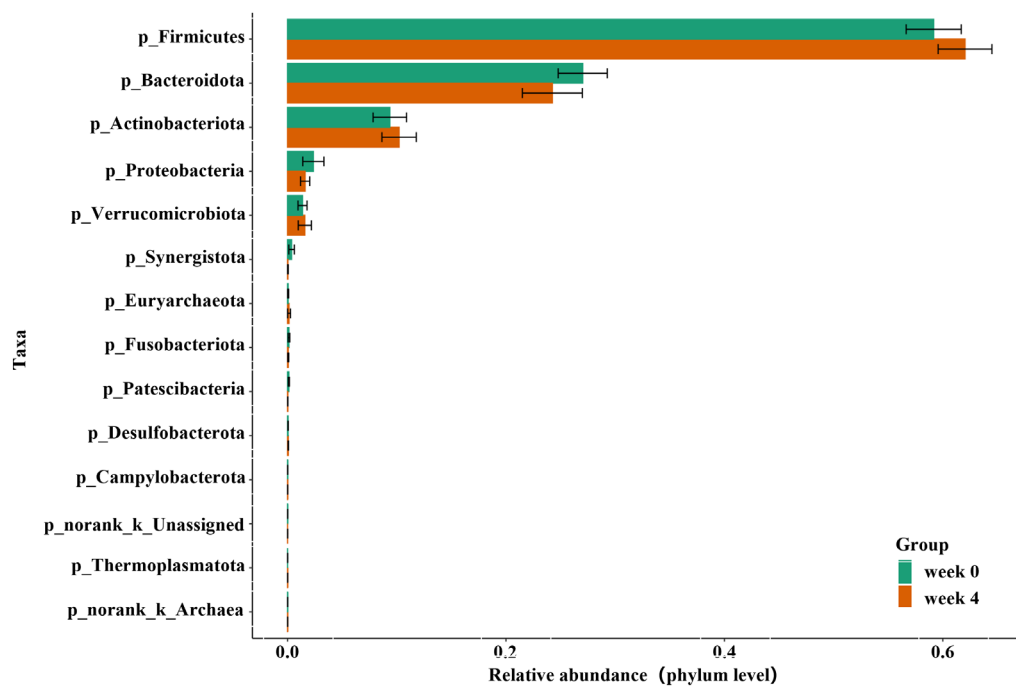

Figure Supplementary Image 1: Relative abundances of intestinal bacteria at the phylum level.

Supplement: Supplementary file 1 [file Image_1.pdf]
